# Combined treatment with CBP and BET inhibitors reverses inadvertent activation of detrimental super enhancer programs in DIPG cells

**DOI:** 10.1038/s41419-020-02800-7

**Published:** 2020-08-21

**Authors:** Maria Wiese, Feda H. Hamdan, Klaudia Kubiak, Christopher Diederichs, Gerrit H. Gielen, Gunther Nussbaumer, Angel M. Carcaboso, Esther Hulleman, Steven A. Johnsen, Christof M. Kramm

**Affiliations:** 1 Division of Pediatric Hematology and Oncology, Department of Child and Adolescent Health, University Medical Center Goettingen, Robert Koch Straße 40 Goettingen, Germany; 2 Gene Regulatory Mechanisms and Molecular Epigenetics Lab, Division of Gastroenterology and Hepatology, Mayo Clinic, 200 First Street SW, Rochester, MN 55905 USA; 3 Institute of Neuropathology, University Hospital Bonn, Venusberg-Campus 1 Bonn, Germany; 4 Division of Pediatric Hematology/Oncology, Department of Pediatrics and Adolescent Medicine, Medical University of Graz, Auenbruggerplatz 38 Graz, Austria; 5 Pediatric Hematology and Oncology, Hospital Sant Joan de Deu/Institut de Recerca, Sant Joan de Deu Barcelona, Spain; 6 Departments of Pediatric Oncology/Hematology, Cancer Center Amsterdam, VU University Medical Centers, Amsterdam, The Netherlands; 7 Princess Máxima Center for Pediatric Oncology, Heidelberglaan 25 Utrecht, The Netherlands; 8 Present Address: Department of Radiation Oncology, Radiooncology/Radiobiology, Klinikum rechts der Isar, Technische Universität München, Ismaninger Str. 22 Munich, Germany

**Keywords:** Paediatric cancer, Preclinical research

## Abstract

Diffuse intrinsic pontine gliomas (DIPG) are the most aggressive brain tumors in children with 5-year survival rates of only 2%. About 85% of all DIPG are characterized by a lysine-to-methionine substitution in histone 3, which leads to global H3K27 hypomethylation accompanied by H3K27 hyperacetylation. Hyperacetylation in DIPG favors the action of the Bromodomain and Extra-Terminal (BET) protein BRD4, and leads to the reprogramming of the enhancer landscape contributing to the activation of DIPG super enhancer-driven oncogenes. The activity of the acetyltransferase CREB-binding protein (CBP) is enhanced by BRD4 and associated with acetylation of nucleosomes at super enhancers (SE). In addition, CBP contributes to transcriptional activation through its function as a scaffold and protein bridge. Monotherapy with either a CBP (ICG-001) or BET inhibitor (JQ1) led to the reduction of tumor-related characteristics. Interestingly, combined treatment induced strong cytotoxic effects in H3.3K27M-mutated DIPG cell lines. RNA sequencing and chromatin immunoprecipitation revealed that these effects were caused by the inactivation of DIPG SE-controlled tumor-related genes. However, single treatment with ICG-001 or JQ1, respectively, led to activation of a subgroup of detrimental super enhancers. Combinatorial treatment reversed the inadvertent activation of these super enhancers and rescued the effect of ICG-001 and JQ1 single treatment on enhancer-driven oncogenes in H3K27M-mutated DIPG, but not in H3 wild-type pedHGG cells. In conclusion, combinatorial treatment with CBP and BET inhibitors is highly efficient in H3K27M-mutant DIPG due to reversal of inadvertent activation of detrimental SE programs in comparison with monotherapy.

## Introduction

Diffuse intrinsic pontine gliomas (DIPG) are pediatric high-grade gliomas (pedHGG) accounting for approximately 10–15% of pediatric central nervous system tumors. Despite major improvements in treatment strategies, the prognosis for DIPG patients persists to be dismal with 5-year survival rates of 2%. Approximately 85% of all DIPGs are characterized by a mutation in the genes encoding either histone 3.3 or 3.1, resulting in a lysine-to-methionine substitution at position 27 (H3K27M)^1^. The H3K27M mutation is supposed to contribute to this aggressive tumor biology, probably by inducing a stem cell-like phenotype^2^.

The H3K27M mutation significantly impairs EZH2 methyltransferase function within the PRC2 complex, and leads to dramatic chromatin changes characterized by a global loss of trimethylation accompanied by hyperacetylation at lysine 27 of histone 3 (H3K27me3)^3^. Acetylation at lysine 27 of histone 3 (H3K27ac) is mediated by the KAT3 family of acetyltransferases (HATs) including CREB-binding protein (CBP). In addition to its function as an acetyltransferase, CBP serves as a protein bridge that connects other transcription factors to the transcription machinery, and acts as scaffold protein during the formation of multicomponent transcriptional regulatory complexes^4^. CBP is necessary for the recruitment of diverse transcription factors, including the Bromodomain and Extra-Terminal (BET) protein BRD4, to genomic regions displaying an enrichment of acetylated nucleosomes, thereby promoting transcription^4–6^. Consequently, BRD4 and CBP are promising pharmaceutical targets for treatment of DIPG and pedHGG^5,6^.

Notably, BET inhibition has been reported to attenuate activation of oncogenic programs in DIPG driven by a subtype of enhancers referred to as “super enhancers” (SE)^7^. SE are clusters of enhancers that are highly enriched for activation factors such as BRD4 and CBP, and are known to drive oncogenic programs in various malignancies^8^. In this study, we aim to evaluate the potential utility of combinatorial BET and CBP inhibition in DIPG. Furthermore, we characterized the effects of these inhibitors on SE programs.

## Materials and methods

### Cell culture

DIPG and pedHGG cell lines were cultured as previously described^9^. SF188 cells were cultured in DMEM/F12. To provide various cellular phenotypes that represent the heterogeneity of DIPG tumor samples and may differentially respond to drug treatment, cells were grown as gliomaspheres in tumor stem cell medium (TSM) or under differentiation conditions in TSM supplemented with 10% FCS, as recommended in ref. ^9^. Unless stated otherwise, cells were treated with 2.5 µM ICG-001 (Calbiochem, Darmstadt, Germany), 2.5 µM PRI-724 (Selleckchem, Munich, Germany), and 0.25 µM (+)/–JQ1 (Selleckchem) dissolved in DMSO.

### Cell viability assays

MTT assays, to monitor x-fold cell growth over time, were carried out as described previously^6^. BrDU assays were conducted using the Cell Proliferation ELISA, BrDU-Kit (Merck, Darmstadt, Germany) according to the manufacturer’s instructions. Crystal violet staining was performed as described previously^10^.

### Clonogenicity assays

For colony- and sphere-formation assays, 2500 cells/ml were seeded in TSM with or without 10% FCS, respectively, and treated with the indicated inhibitors. Gliomaspheres were stained as previously described^6^, scanned, and analyzed using the particle analyzer plugin from ImageJ^11^.

### Migration and invasion assays

Migration and invasion assays had been performed and analyzed as previously described^6^. Briefly, 5000 cells of 48-h pretreated cells in FGF-depleted TSM-work medium were used, and migration was analyzed after 24 h.

### Quantitative real-time PCR (qPCR) and western blotting

RNA was extracted using the ReliaPrep^TM^ RNA Cell Miniprep System (Promega, Walldorf, Germany) according to the manufacturer’s instructions. cDNA synthesis followed by qPCR with the PowerUp SYBR Green Mastermix (Thermo Fisher Scientific, Osterode am Harz, Germany) and western blotting was performed as previously described^6,12^. Oligonucleotides and antibodies can be found in the supplemental materials and methods.

### Chromatin immunoprecipitation followed by next-generation sequencing

ChIP-seq was performed as previously described^13^. Briefly, cells were cross-linked in 1% formaldehyde followed by quenching with glycine (final concentration 125 mM), lysed, and sonicated for 30 cycles (30 s on/30 s off) (Bioruptor pico, Diagenode, Liege, Belgium). Precleared chromatin was incubated with 1 µg of antibody (for antibodies see supplemental materials and methods). Protein A-sepharose beads were used to pull down the antibody–chromatin complex, and samples were then de-cross-linked, and DNA was extracted.

### Library preparation and next-generation sequencing

Libraries from RNA were synthesized using the TruSeq RNA Library Prep Kit v2 (Illumina, Munich, Germany) according to the manufacturer’s instructions. Microplex Library preparation kit v2 (Diagenode) was used to prepare libraries from ChIP DNA. Pools of libraries were sequenced in the Transcriptome and Genome Analysis Laboratory (TAL) at the University Medical Center Göttingen using HiSeq4000 (Illumina, 50SE).

### Bioinformatic analysis

Primary ChIP and RNA-seq analysis were performed as in ref. ^13^. SEs were called using the ROSE algorithm^8^ by sorting enhancers based on the H3K27ac signal and using default settings with ignoring regions within 2.5 kb of annotated TSS. Associated genes were identified using GREAT analysis, and enriched factors at SE regions were identified using ReMAP^14,15^. Gene set enrichment analysis was performed using FPKM values of expressed genes in various conditions with SE selected as a text entry-based gene database. Clustering of gene expression patterns of SE-associated genes was performed using short time-series expression miner (STEM).

## Results

### H3.3K27M-mutated DIPG cells display strong stem-like potential and proliferation activity compared with H3WT-pedHGG cells

**Fig. 1:**
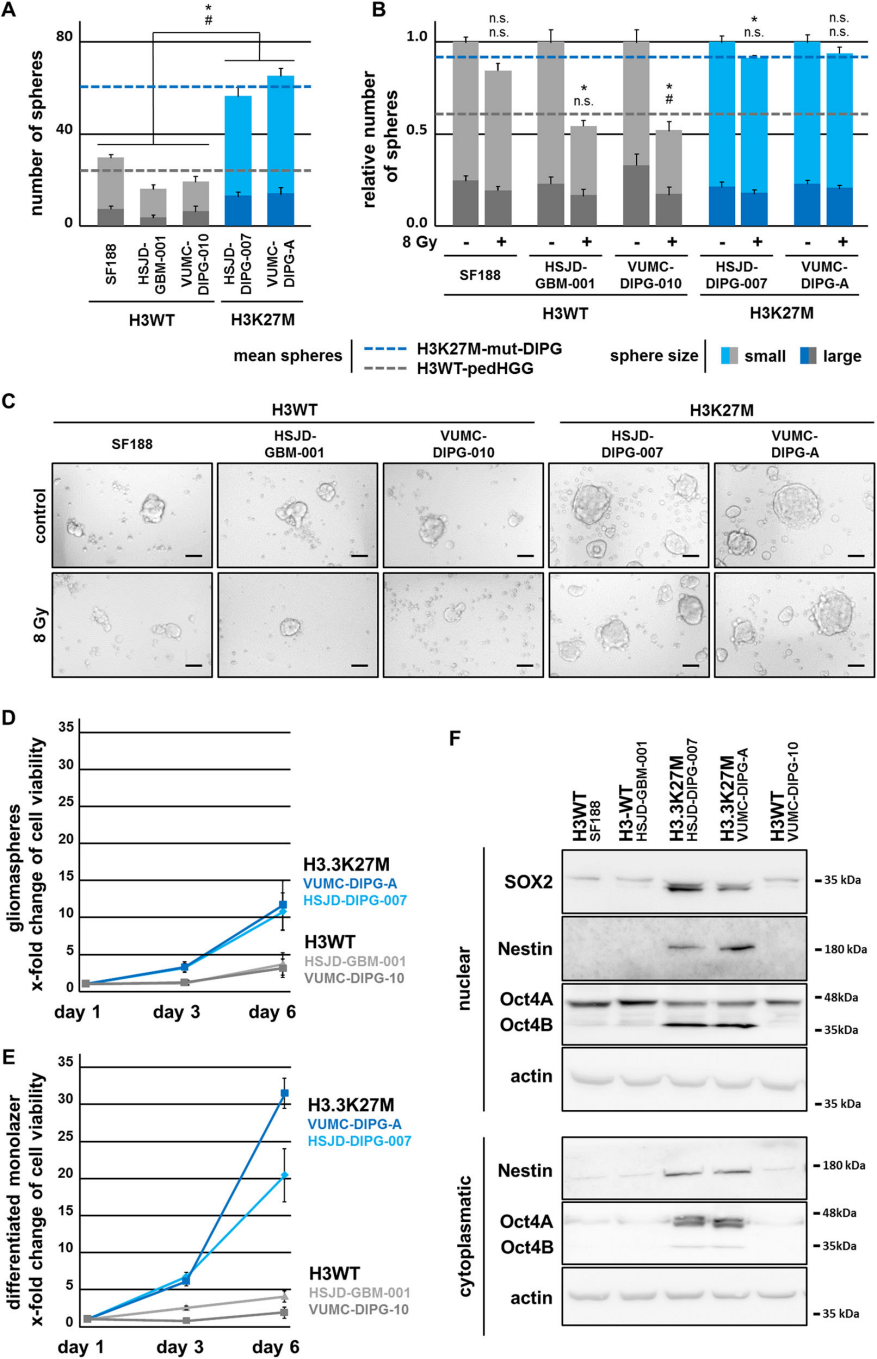
H3.3K27M-mut-DIPG cells show higher proliferation, stem cell-like characteristics, and resistance to irradiation in comparison with H3WT-pedHGG cells. **a** Total number of H3WT and H3.3K27M-mut gliomaspheres after 5 days. **b** Quantification and (**c**) bright-field images of H3WT and H3.3K27M-mut gliomasphere formation after irradiation with 8 Gy (after 24 h) after 5 days. Spheres where scored according to their size: large (>0.1 mm) and small (<0.1 mm), # large and Δ small spheres, scale bar 1 mm. **d**, **e** Cell viability assessed by MTT assay, reflecting the cell growth over time of primary H3.3K27M-mut-DIPG and H3WT-pedHGG/DIPG cells grown under stemness- (gliomaspheres) and differentiation- (monolayer) conditions. **f** Protein expression of stemness-associated markers Oct4, Nestin, and Sox2 in cytoplasmatic and nuclear protein fractions of H3WT and H3.3K27M gliomaspheres, assessed by western blotting, β-actin served as loading control.

To evaluate if H3K27M mutation leads to changes in cell growth in vitro, we investigated the potential of H3 wild-type (H3WT) and H3K27M-mut pedHGG and DIPG cell lines to form gliomaspheres. In accordance with previous reports demonstrating that H3K27M mutation confers a more stem cell-like phenotype^2^, we observed a stronger potential of the H3.3K27M-mutant VUMC-DIPG-A and HSJD-DIPG-007 cell lines to form gliomaspheres under stem cell conditions. In contrast, spheroid formation of the primary DIPG cell line VUMC-DIPG-10 and the pedHGG cell lines SF188 and HSJD-GBM-001 harboring H3WT was less efficient (Fig. 1a, c). Consistently, we observed a strong expression of the stem cell markers Oct4, Sox2, and Nestin in H3.3K27M-containing DIPG cell lines. Conversely, expression was reduced or absent in H3WT-pedHGG and H3WT-DIPG gliomasphere cell lines (Fig. 1g). The expression of Oct4 and Sox2 was strongly reduced in all monolayer cells after culture under differentiation conditions for 3 days, but still slightly more expressed in H3.3K27M-mut-DIPG in comparison with H3WT-pedHGG cells (Supplemental Fig. 1).

Interestingly, H3.3K27M-mut-DIPG cells, grown either as a differentiating monolayer or in stem cell-like gliomaspheres, displayed a higher proliferation rate than H3WT-pedHGG/DIPG cells (Fig. 1d, f and Supplemental Fig. 2B). The higher proliferation potential of H3.3K27M-mut-DIPG was not accompanied by a stronger susceptibility to irradiation in comparison with primary H3WT-pedHGG cells. On the contrary, sphere formation of H3.3K27M-mut-DIPG cells was almost unaffected by irradiation, indicating an additional mechanism of resistance in H3.3K27M-mutant DIPG, which is missing in H3WT-pedHGG/DIPG (Fig. 1b, c). These findings indicate the importance of H3K27M mutation for tumor phenotype and resistance to radiotherapy in H3.3K27M-mutated DIPG.

### Combinatorial inhibition of BET and CBP activity markedly affects proliferation of H3.3K27M-mut-DIPG cells

**Fig. 2:**
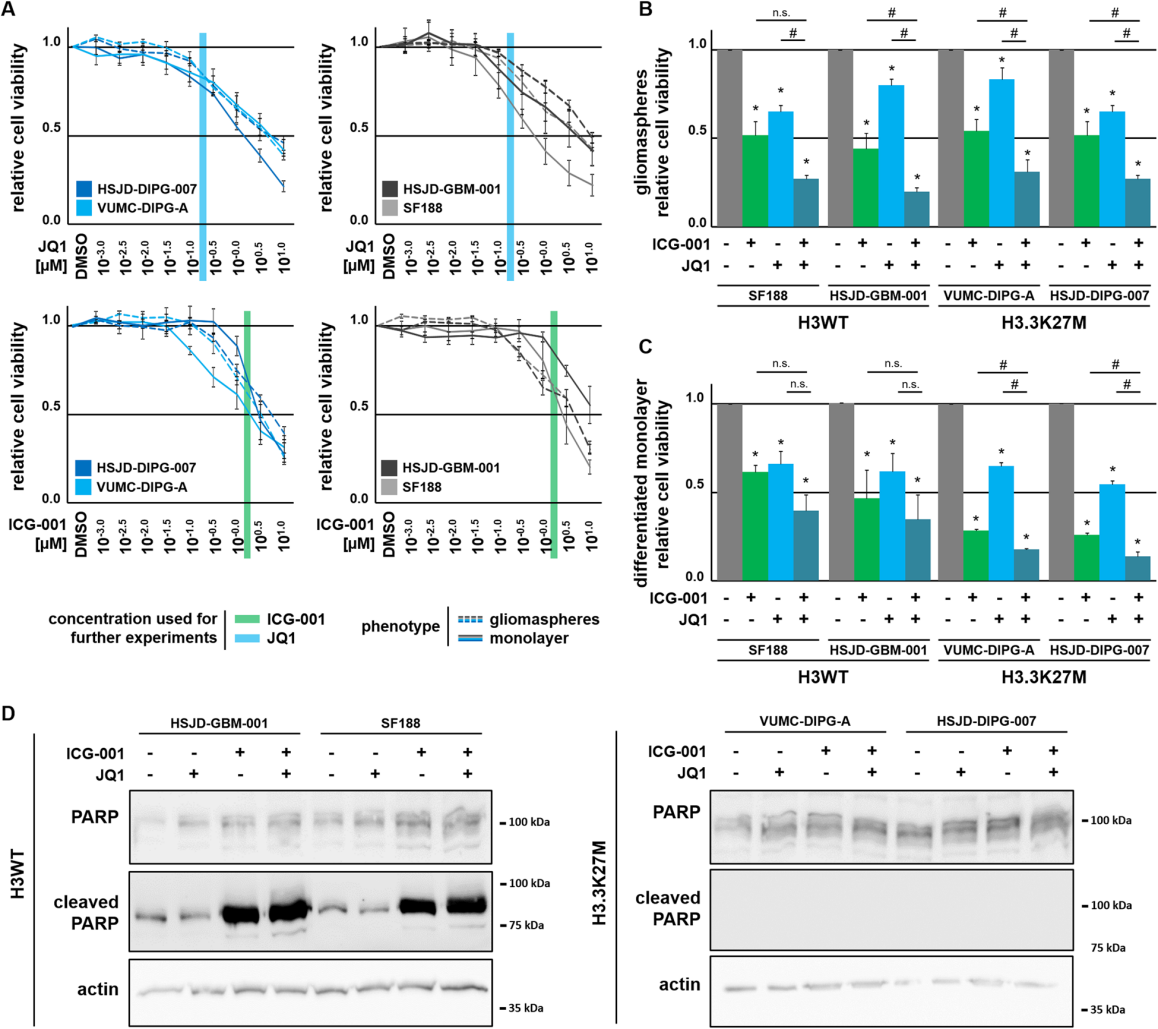
Cell viability is strongly dependent on CBP and BET function. **a** MTT cell viability dilution curves with increasing concentrations of JQ1 and ICG-001, as indicated. H3K27M-mut-DIPG cells were treated with ICG-001 and JQ1, as indicated and subjected to MTT cell viability assay with H3K27M-mut-DIPG cells grown under (**b**) stemness- (gliomaspheres) and (**c**) differentiation- (monolayer) conditions. *p < 0.05 with respect to DMSO-treated control cells, #*p* < 0.05 with respect to ICG-001 or JQ1-treated cells, as indicated. **d** Protein expression of cleaved PARP apoptosis marker in H3WT-pedHGG and H3K27M-mut-DIPG cells after ICG-001 and JQ1 treatment, as indicated; β-actin served as loading control.

Given the proposed importance of enhancer activation in DIPG, we next tested the effects of inhibition of CBP or BET proteins. Cell viability of H3.3K27M-mut-DIPG and H3WT-pedHGG gliomaspheres and monolayer cells was reduced upon CBP or BET protein inhibition in a dose-dependent manner (Fig. 2a). Combined treatment with the CBP inhibitor ICG-001 and the BET inhibitor JQ1 led to the strongest effects in monolayer cells of H3.3K27M-mut-DIPG and H3WT-pedHGG gliomaspheres. Notably, additive effects of ICG-001 and JQ1 were observed in gliomaspheres, irrespective of the H3 mutation status (Fig. 2b, c). Comparison of cell quantification by crystal violet staining, proliferation by BrdU incorporation, and MTT analyses confirmed the reduced cell viability following BET and CBP inhibition (Supplemental Fig. 2A, B). Interestingly, JQ1 alone or in combination with ICG-001 induced apoptosis in H3WT-pedHGG cells as determined by PARP cleavage. Notably, in spite of their similar response to treatment, no apoptosis was observed in H3.3K27M-mut-DIPG cells.

### Combinatorial inhibition of CBP and BET proteins attenuates tumor-associated characteristics of H3.3K27M-mut-DIPG

**Fig. 3:**
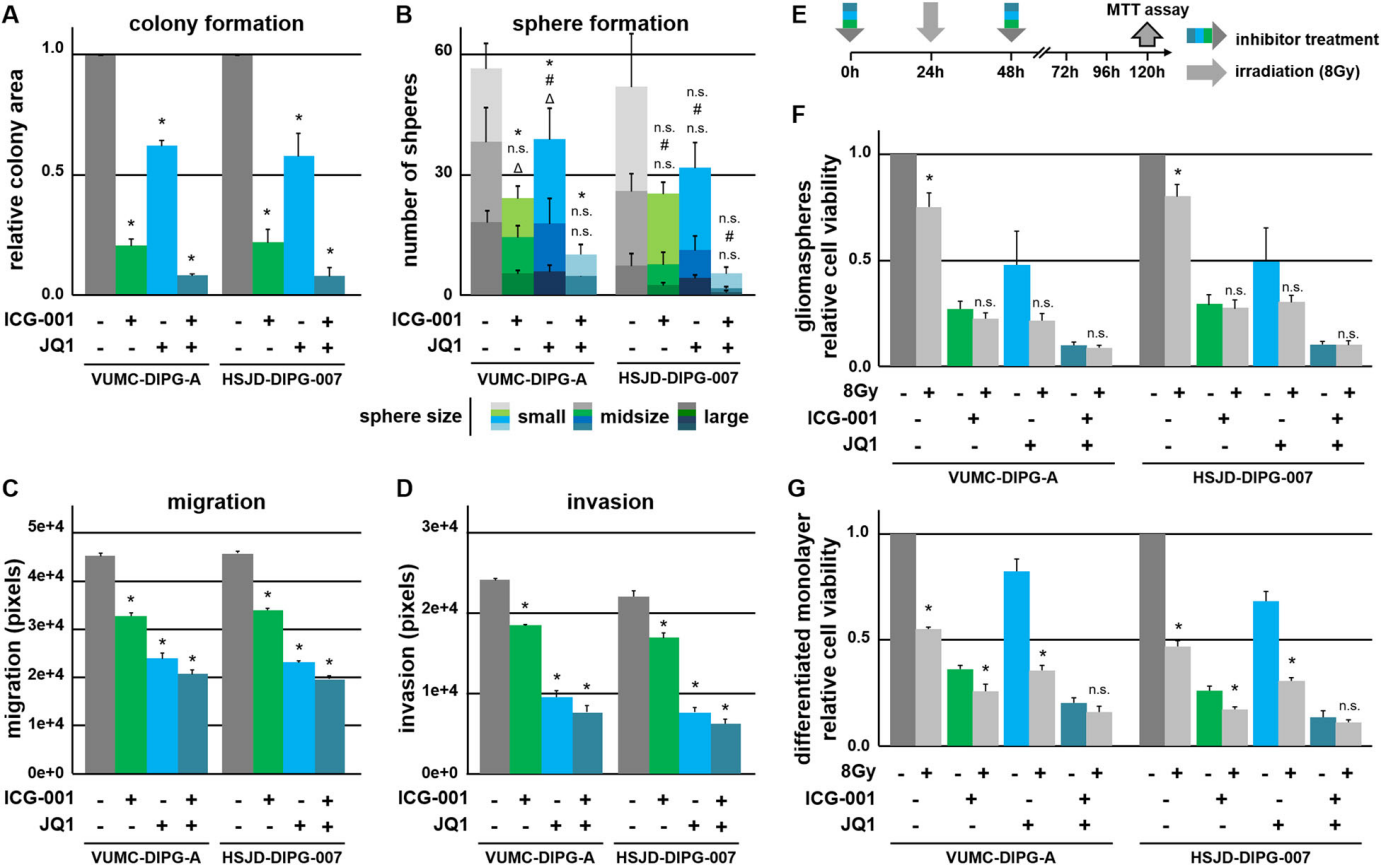
Combined treatment with JQ1 and ICG-001 results in strong cytotoxic effects and significantly suppresses tumor-associated characteristics of H3K27M-mut-DIPG cells. **a** Colony-formation assay (*p < 0.05) and (**b**) sphere-formation assay showing the total number of gliomaspheres, scored according to their size: large (>0.1 mm), mid-sized (0.1–0.3 mm), and small (0.05–0.1 mm), *p* *<* *0.05* for * large, # midsize, and Δ small spheres. **c** Migration and (**d**) invasion of H3K27M-mut-DIPG cells after treatment with ICG-001 and JQ1, as indicated. (**e**–**g**) MTT cell viability assays of H3K27M-mut-DIPG cells grown under (**f**) stemness- (gliomaspheres) and (**g**) differentiation- (monolayer) conditions after ICG-001 and JQ1 treatment (at days 1 and 3) in combination with 8 Gy irradiation (at day 2) after 6 days of total treatment, (**e**) as schematically shown (DMSO control, 0 Gy = 100%, *p < 0.05 with respect to similarly treated, nonirradiated cells).

Given their marked effect on proliferation, we further investigated the function of ICG-001 and JQ1 in H3K27M-mut-DIPG cells. BRD4 was shown to be essential for maintaining stemness characteristics in gastric cancer and embryonic stem cells^16,17^. Due to high spheroid-forming potential in H3.3K27M-mut-DIPG, we tested if BET and CBP inhibition exerts inhibitory effects on these tumor-associated characteristics. Indeed, colony- and sphere-formation assays demonstrated decreased self-renewal potential and proliferation upon treatment with ICG-001 and/or JQ1 (Fig. 3a, b). DIPG are also characterized by strong migration and invasion potential. Notably, inhibition of CBP decreased migration and invasion, while BET inhibition affected both to an even greater extent (Fig. 3c, d). Since radiotherapy is widely regarded as standard treatment for DIPG, we further tested whether BET and CBP inhibition may increase the radiosensitivity of DIPG cells (Fig. 3e–g). As expected, irradiation was most effective in differentiated monolayer DIPG cells in comparison with more slowly proliferating gliomaspheres (Fig. 3e, f and Fig. 1c, d). This phenotype is further associated with mechanisms that are protective against radiation-induced DNA damage^18^. Remarkably, combined therapy attenuated cell growth to an extent comparable to radiotherapy alone. Moreover, BET inhibition in DIPG gliomaspheres led to a stronger susceptibility to irradiation (Fig. 3f, g), which was supported by a strong reduction in self-renewal (Fig. 3b). These observations further demonstrate the positive effect of combinatorial inhibition of BET and CBP for DIPG treatment, especially after a prolonged incubation time of overall 5 days.

### Identification of super enhancer programs in DIPG cells

**Fig. 4:**
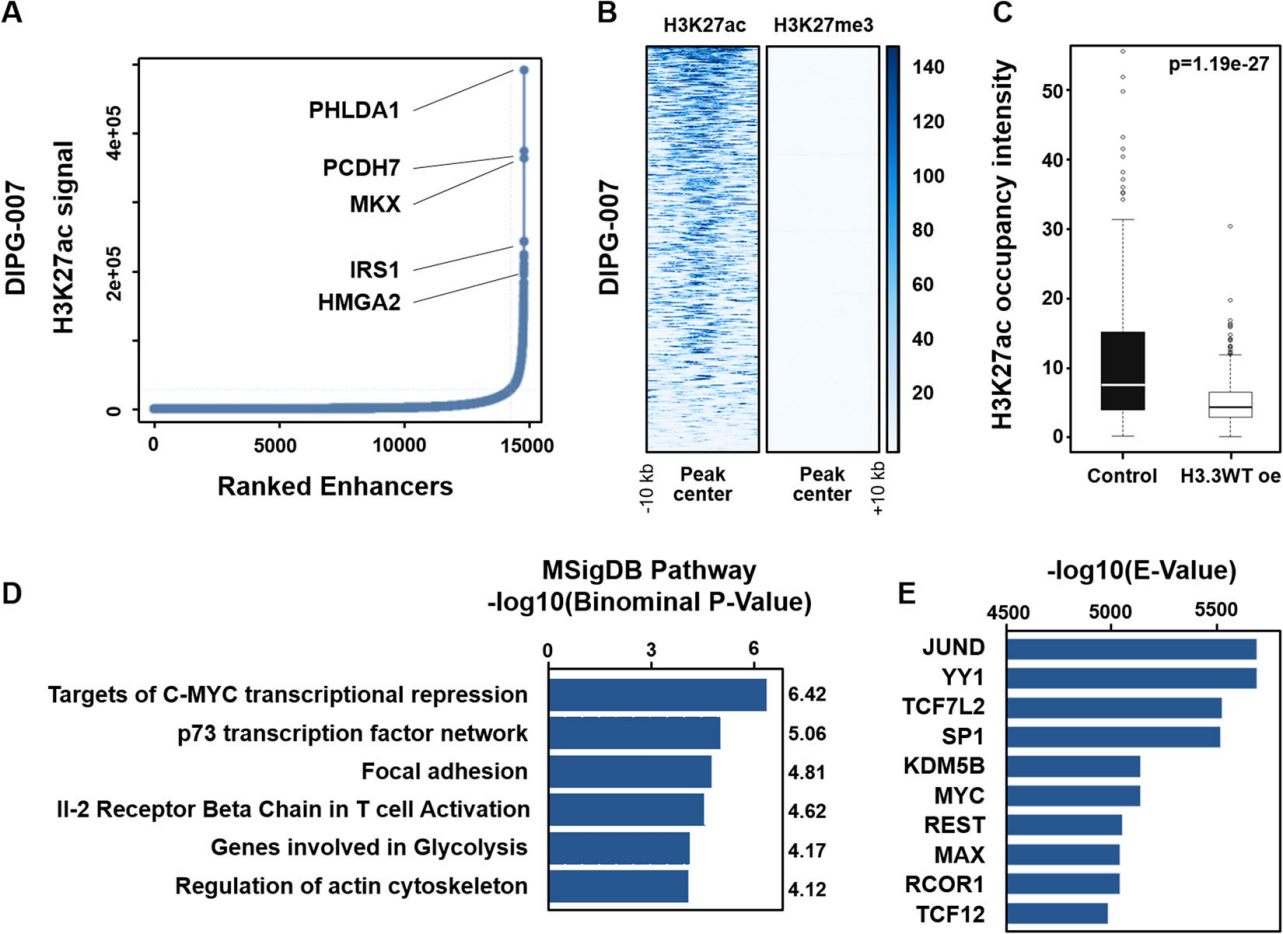
Identification of super enhancer programs in H3.3K27M DIPG cells. **a** Output of the ROSE algorithm depicting ranking of enhancer regions based on H3K27ac after stitching with individual example genes shown. **b** Affinity profile plot for H3K27ac at super enhancer regions showing high occupancy of H3K27ac and no occupancy of H3K27me3. **c** Box plot of H3K27ac mean intensity at super enhancer regions (±5 kb) from publicly available data in control DIPG-007 cells and DIPG-007 with overexpression of wild-type H3.3. **d** Pathway enrichment performed by GREAT analysis on genes associated with super enhancers showing programs activated in DIPG-007. **e** ReMAP analysis output identifying the factors that most significantly nucleate super enhancer regions in DIPG-007.

SE have previously been implicated in tumor progression and aggressiveness in DIPG^7^. However, little is known about the SE landscape in H3.3K27M cells. Accordingly, we performed ChIP-seq for H3K27ac and H3K27me3 in DIPG-007 cells and identified SE regions (Fig. 4a). As expected, these regions were highly occupied by H3K27ac and were devoid of H3K27me3 (Fig. 4b). Comparison of the identified SE with published data from DIPG-007 cells with ectopic H3WT overexpression^19^ confirmed that the identified SE regions are significantly dependent upon H3K27M in DIPG (Fig. 4c). Pathway enrichment analysis for genes associated with these SE revealed a c-MYC signature, which is in concordance with previous reports highlighting the role of MYC in DIPG (Fig. 4d)^20,21^. Additional enriched pathways included p73, which has previously been associated with a more invasive signature in glioblastomas^22^. To further characterize these SE, we used ReMAP to identify transcription factors that may potentially nucleate these regions (Fig. 4e). Interestingly, MYC and MYC-associated factor X (MAX) were identified among the most significantly enriched factors. Other transcription factors included the AP1 factor JUND, which has been found to mediate detrimental tumor biological effects in glioblastomas^23^. In addition, Transcription Factor 7 Like 2 (TCF7L2), which directly interacts with CBP^24^, was found to be highly enriched at these SE regions. In general, identification of SE in the DIPG-007 can uncover potential therapeutic targets and pathways that can be leveraged to increase survival in DIPG patients.

### Activation of a subset of SE by inhibition of CBP or BET can be reversed by combined therapy

**Fig. 5:**
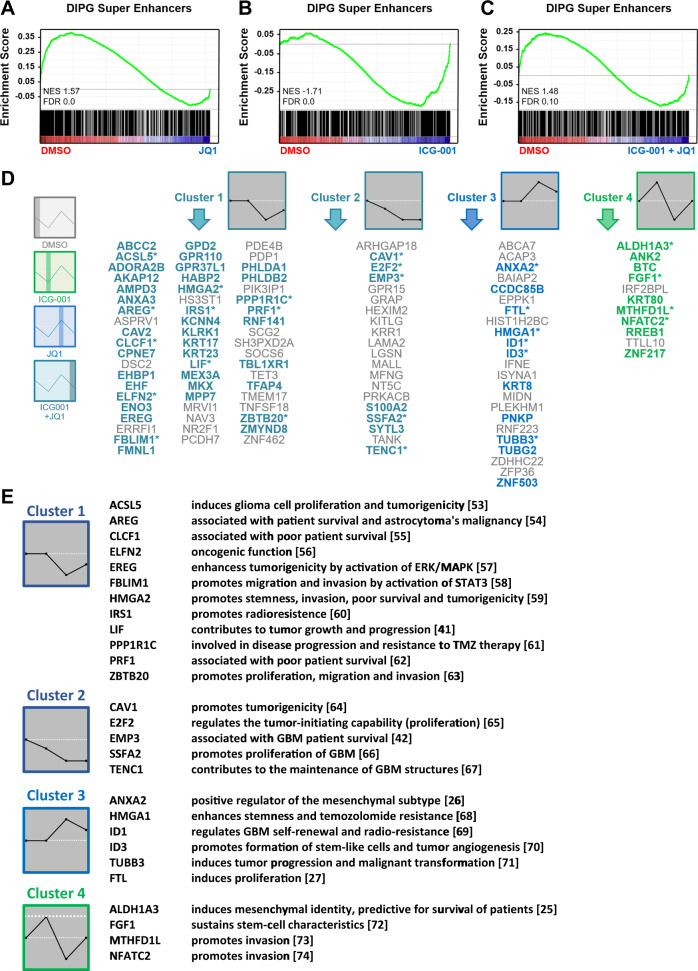
CBP and BET inhibition downregulates super enhancer programs inadvertently activated by individual therapies. **a**–**c** GSEA plots showing enrichment of super enhancer programs in control compared with treatment (**a**) of JQ1, (**b**) ICG-001, and (**c**) combinatorial treatment. **d** Significant clusters showing different patterns of gene expression regulation upon treatment with DMSO, JQ1, ICG-001, or combined ICG-001/JQ1 treatment. Asterisks mark genes that promote GBM characteristics. Bold and colored genes promote other tumor entities. **e** Genes of four clusters differentially regulated by ICG-001, JQ1, and combinatorial treatment and their function in GBM pathogenesis^52–73^.

**Fig. 6:**
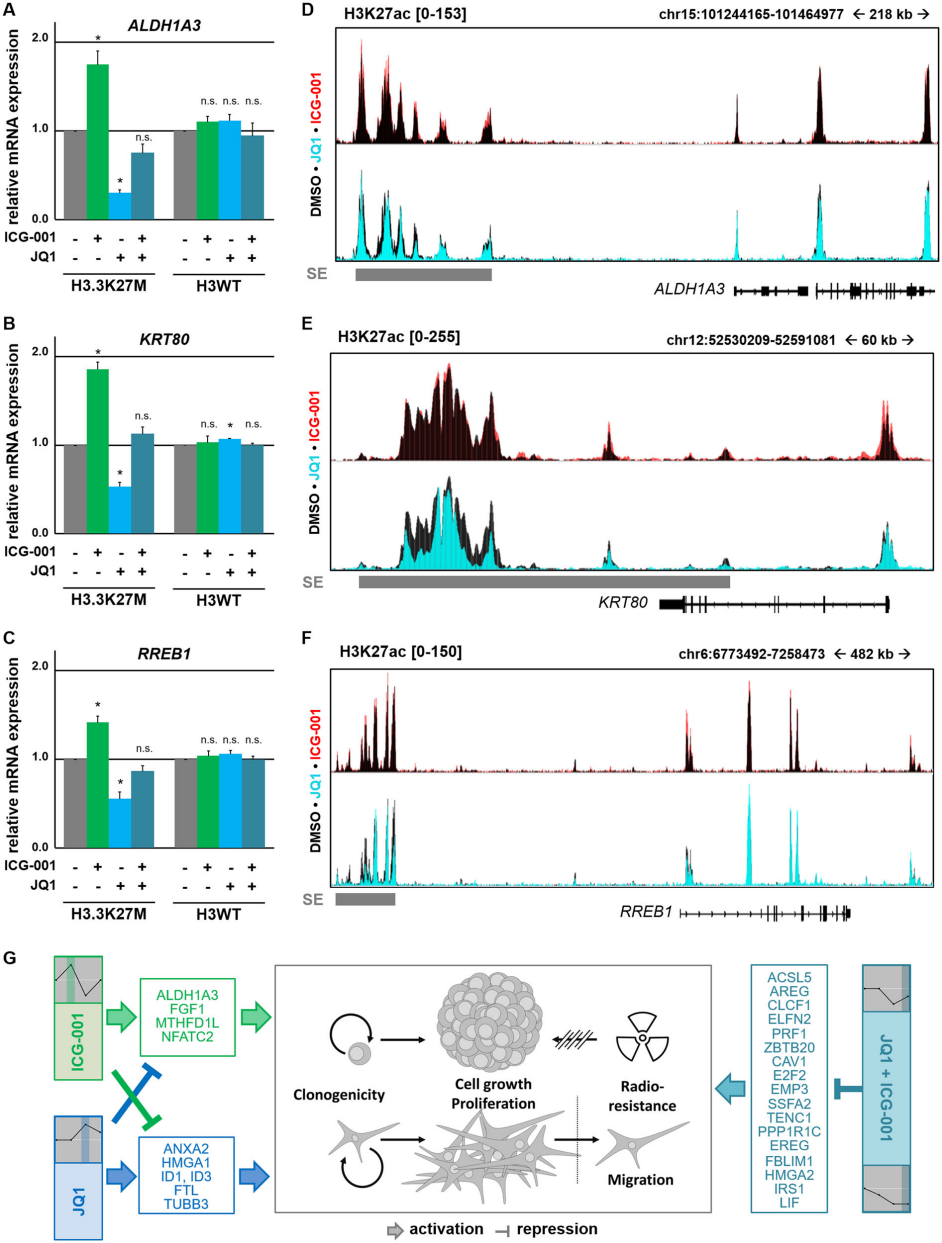
Inadvertently activated gene expression in DIPG-007 cells by single treatment with ICG-001 and JQ1 is reversed by combined treatment due to inhibition of associated super enhancers. **a**–**c** qPCR validating the gene expression of (**a**) ALDH1A3, (**b**) KRT80, and (**c**) RREB1 in DIPG-007 and GBM-001 cells after 48 h of treatment with ICG-001 and JQ1, as indicated. *P* < 0.05. **d**–**f** Occupancy profiles of H3K27ac at ALDH1A3, KRT80, and RREB1 in cells treated with JQ1 or ICG-001 overlaid on DMSO-treated cells for comparison. Super enhancers in control cells are shown in gray. **g** Graphical model depicting the mutual effect of JQ1 and ICG-001 on detrimental programs affecting each other and leading to an increased effect of combinatorial therapy.

As BET inhibition was extensively documented to specifically downregulate SE programs in various cancer types including DIPG^7,8^, we hypothesized that the combinatorial effects we observed with BET and CBP inhibition may be due to potentiated effects in silencing the SE programs. Accordingly, we performed RNA- and ChIP-seq analyses from cells treated with JQ1, ICG-001, or their combination. As expected, GSEA analysis showed that JQ1 treatment significantly downregulated the majority of genes associated with SE (Fig. 5a). In contrast, analysis in ICG-001-treated cells revealed that most genes associated with SE in DIPG-007 cells were significantly upregulated (Fig. 5b). Interestingly, combined therapy led to a less-weighted pattern of regulation with half of the genes observed to be upregulated compared with DMSO and the other half downregulated (Fig. 5c). To further characterize the altered patterns of regulation in combined therapy, we clustered the gene expression changes in SE-associated genes using the STEM algorithm and identified four significant clusters (Fig. 5d). Cluster 1 included 58 genes that were specifically downregulated by BET inhibition and unperturbed by CBP inhibition whether alone or in combination. These genes included many that are known to be associated with a more aggressive and invasive phenotype in various cancer types including glioblastoma. Cluster 2 included 20 genes that are downregulated in all conditions. Importantly, cluster 3 included 23 genes that were upregulated by JQ1 and downregulated by ICG-001. Surprisingly, genes in this cluster were associated with an unfavorable prognosis in glioma, like Annexin A2 (*ANXA2*)^25^ and Ferritin Light Chain (*FTL*)^26^. Interestingly, the fourth cluster comprised 11 genes whose upregulation by ICG-001 was prevented by concomitant treatment with JQ1. This cluster also included detrimental genes that are associated with poor survival and invasion, such as Aldehyde Dehydrogenase 1 family member A3 (*ALDH1A3*)^27,28^, Keratin 80 (*KRT80*)^29^, and Ras Responsive Element Binding Protein 1 (*RREB1*)^30^ (for summary see Fig. 5e). qPCR analyses of H3K27M-DIPG-007 and H3WT-HSJD-GBM-001 cells confirmed that these genes are upregulated by CBP inhibition, but this activation is reversed by combinatorial treatment due to high sensitivity to BET inhibition (Fig. 6a–c). The same pattern of activation was observed for the super enhancers associated with these genes as confirmed by ChIP-seq following inhibition of BET and CBP monotherapy (Fig. 6d–f). These observations point to the activation of detrimental super enhancers by single treatment with either JQ1 or ICG-001, whereas combined treatment reverses these inadvertent programs (for summary see Fig. 6g).

## Discussion

DIPG are the most aggressive brain tumors among pediatric high-grade glioma with median survival rates of less than 1 year. Due to their diffuse growth and location, DIPG are inoperable, and the current therapeutic gold standard remains to be radiation therapy (RT) that confers only a few months survival advantage. In 2012, identification of frequently occurring H3K27M mutations in up to 85% of all DIPG uncovered a shift of the epigenetic balance reflected by global loss of H3K27me3^1^. Interestingly, H3.3K27M in DIPG is not associated with an unfavorable prognosis compared with DIPG carrying H3WT, indicating that additional factors may contribute to the poor prognosis in DIPG^1^. In contrast, survival differences are observed between H3K27M mutant and H3 wild type in non-pontine diffuse midline gliomas^31^. This suggests that H3.3K27M mutation might play a more prominent role in non-pontine midline gliomas compared with DIPG. However, H3K27M-mutated DIPG cell lines appear to behave in general more aggressively in vitro compared with H3WT-DIPG and pedHGG cells confirming that additional factors contribute to the poor survival of DIPG patients. H3K27me3-deficient nucleosomes in H3K27M-mut-DIPG acquire H3K27ac, resulting in the formation of H3K27M-K27ac heterotypic nucleosomes and global hyperacetylation of H3K27.

Hyperacetylation favors the function of transcriptionally active, acetylation-dependent factors such as BET proteins^5^. Previous investigations showed that BET inhibition results in DIPG growth suppression in vitro and in vivo^5,7,32,33^. Consistently, we showed that H3.3K27M-mut-DIPG cells are highly sensitive to JQ1. Moreover, gene expression profiling in H3.3K27M-mut-DIPG cells revealed that many oncogenic programs are attenuated by JQ1 treatment. On the other hand, we hypothesized that acetyltransferases such as CBP may play a significant role in the context of DIPG malignancy as it was reported to highly occupy super enhancers^34^. Indeed, H3WT-pedHGG as well as H3.3K27M-mut-DIPG cell growth was sensitive to CBP inhibition. Interestingly, monolayer cultures of H3.3K27M-mut-DIPG cells grown under differentiation conditions showed a stronger response to treatment with ICG-001. Notably, CBP was shown to be crucial for the activation of genes that promote normal differentiation of cortical precursors into neuronal, astrocytic, and oligodendroglial precursors^35^. However, the particular function of CBP in this context in DIPG needs to be further examined.

Moreover, we further investigated the combined effect of CBP and BET inhibition on H3.3K27M-mut-DIPG cells. Surprisingly, we found that the combinatorial effect of JQ1 and ICG-001 efficiently hinders proliferation, sphere formation, and radioresistance with strongest effects after combinatory treatment. This was not expected given the relatively moderate effects after monotherapy. Determination of the expression changes and affected SE by single and combinatorial use of BET and CBP inhibitors revealed partially antagonistic and detrimental effects caused by ICG-001 or JQ1, which were eliminated after combination of these inhibitors. As expected, JQ1 treatment significantly downregulated most genes associated with SE, which we identified in H3.3K27M-mut-DIPG cells. In contrast, we observed that monotherapy with ICG-001 upregulates the majority of SE-associated genes. This effect is quite surprising, given the reported enrichment of CBP as a feature for SE^36^. However, ICG-001 does not inhibit CBP catalytic activity, but instead blocks interaction with other proteins such as β-catenin and RXR^37^. Consequently, it is possible that inhibition of such interactions may facilitate others, thereby promoting the nucleation of SE driven by factors whose interaction with CBP is not inhibited by ICG-001. Thus, it would be of interest to evaluate the effects of newly developed CBP inhibitors that exhibit high selectivity and target the catalytic region of CBP^38^. In addition, we uncovered that JQ1 and ICG-001 upregulate genes that are associated with a more aggressive phenotype. Importantly, this effect is attenuated when these agents are used in combination.

A similar effect, where the expression of genes upregulated by one agent is blocked by BET inhibition, has already been reported for JQ1 treatment in conjunction with histone deacetylase inhibitors^7,39^. In general, this provides a rational basis for further investigation of combined ICG-001/BET inhibitor treatment as a potential effective therapeutic approach in DIPG.

Interestingly, we identified four different, significant gene sets by clustering the observed gene expression changes in SE-associated genes after single and combined inhibition of CBP and BET proteins. Cluster 1 included downregulated genes by JQ1 and in combination with ICG-001. Cluster 2 included downregulated genes in all conditions. Genes identified in these two clusters are associated with a more aggressive and invasive phenotype in various cancer types including glioblastoma. For example, Leukemia inhibitory factor (LIF) in Cluster 1 that contributes to GBM tumor growth and progression^40^ or Epithelial membrane protein 3 (EMP3) in Cluster 2 whose high expression was shown to be associated with worse GBM patient survival^41^. Genes of Cluster 3 were upregulated by JQ1 and downregulated by ICG-001, and Cluster 4 included genes whose upregulation by ICG-001 was prevented by concomitant treatment with JQ1. Surprisingly, genes in clusters 3 and 4 were associated with an unfavorable prognosis in glioma. For example, Annexin A2 (*ANXA2*), Ferritin Light Chain (*FTL*), and Inhibitor of DNA-binding-1 (*ID1*) fulfill important tumor functions in glioma and glioblastoma, such as the promotion of invasion and tumor progression^42^, proliferation^26^, or chemoresistance^43–45^. The fourth cluster included detrimental genes such as *ALDH1A3* promoting stemness-like features in glioma, and is associated with worse survival^27,28^. Another example, *KRT80*, is overexpressed in more aggressive glioma^46^ and, together with CBP, contributes to therapy resistance^47^, whereas *RREB1* promotes an invasive phenotype^30,48^. In agreement with the function of these genes, ICG-001 treatment of H3.3K27M-mut-DIPG was not as efficient in inhibiting migration and invasion as JQ1 treatment. Interestingly, gene expression profiling upon BET and CBP inhibition revealed that this effect appears to be particularly specific to H3K27M-mut-DIPG cells.

Accordingly, we propose a model where JQ1 and ICG-001 not only inactivate SE-related programs when given alone, but also inadvertently activate detrimental programs that can be attenuated when both agents are used in combination. However, further investigations are needed to elucidate if JQ1, ICG-001, or similar drugs with the same function can pass the blood–brain barrier (BBB) and inhibit DIPG growth in vivo. Recent studies have already successfully proved the BBB permeability of JQ1^49,50^. Although ICG-001 has not been used to treat brain malignancies so far, its derivative PRI-724 has already been investigated in several clinical trials in other types of cancer, including myeloid malignancies, pancreatic cancer, and advanced solid tumors (NCT01302405, NCT01606579, and NCT01764477). Notably, PRI-724 leads to comparable effects to those seen with ICG-001 in DIPG and pedHGG cells (Supplemental Fig. 3A, B). ICG-001 had been additionally found to transiently increase BBB permeability due to depression of endothelial cell–cell interaction^51^.

In conclusion, the combinatorial use of JQ1 and ICG-001 showed a high efficacy in attenuating the growth and self-renewing potential of DIPG cells. In addition, JQ1 and ICG-001 exhibited an antiproliferative effect that is comparable to radiation alone. We report that ICG-001 and JQ1 inadvertently activate a subgroup of detrimental super enhancer programs that are reversed in combination.

## Supplementary information

supplemental Tables

Figure S1

Figure S2

Figure S3

Supplementary Figures legends

## Data Availability

Raw datasets for ChIP/RNA-seq were deposited in ArrayExpress (https://www.ebi.ac.uk/arrayexpress/) under the accession numbers E-MTAB-8242 for ChIP-seq, E-MTAB-9152 and E-MTAB-8243 for RNA-seq.
