# Supplementary material for: Combined treatment with CBP and BET inhibitors reverses inadvertent activation of detrimental super enhancer programs in DIPG cells
Source: Cell Death Dis. 2020 Aug 21;11(8):673. doi: 10.1038/s41419-020-02800-7 (PMC7442654; doi:10.1038/s41419-020-02800-7)
Supplement: Supplementary file 1 — supplemental Tables [file 41419_2020_2800_MOESM1_ESM.docx]

**Supplemental Materials and Methods**

Wiese and Hamdan et al.: Combined treatment with CBP and BET inhibitors reverses inadvertent activation of detrimental super enhancer programs in DIPG cells

**Supplemental Materials**

**Supplemental Table 1│ Oligonucleotides used in this study for qPCR**

| **Oligo target** | **Oligo sequence (5’-3’) forward** | **Oligo sequence (5’-3’) reverse** |
| --- | --- | --- |
| *HPRT* | TATGCTGAGGATTTGGAAAGG | CATCTCCTTCATCACATCTCG |
| *KRT80* | TGTGACCAGAGCAACCAGAG | CTGAGGCTGCTGAAGCCAA |
| *RREB1* | TTGCATCTCTCCTCGGTCTC | GAACACACAGTCGGAGCAAC |
| *ALDH1A3* | ACCATCCCCACAGATGACAAC | GGACCATGGTGTTCCCACAG |

**Supplemental Table 2 │ Antibodies used in this study for western blotting**

| **Antibody** | **Order ID** | **Supplier (location)** |
| --- | --- | --- |
| β-actin | A3854 | Sigma-Aldrich (Taufkirchen, Germany) |
| Nestin | 31373 | Acris (Herford, Germany) |
| Sox2 | 3579S | Cell Signaling (Frankfurt, Germany) |
| Oct4 | 0421 | DCS (Hamburg, Germany) |
| cleaved PARP | 9541 | Cell Signaling (Frankfurt, Germany) |
| PARP | 9542 | Cell Signaling (Frankfurt, Germany) |
| H3K27ac | 196-050 | Diagenode (Liege, Belgium) |
| H3K27me3 | C15410195 | Diagenode (Liege, Belgium) |
| rabbit IgG | C15410206 | Diagenode (Liege, Belgium) |

**Supplemental methods**

***Statistics and general methods***

All data are represented as mean of at least three independent biological replicates +/- SEM. P < 0.05 was considered as significant with respect to DMSO treated control cells. Each single experiment was performed in three technical replicates. Except for qPCR and untreated sphere formation showing two-sided, one-sided testing was used for p-value calculation using student's t-tests.
