# Supplementary figures and images for: Combined treatment with CBP and BET inhibitors reverses inadvertent activation of detrimental super enhancer programs in DIPG cells
Source: Cell Death Dis. 2020 Aug 21;11(8):673. doi: 10.1038/s41419-020-02800-7 (PMC7442654; doi:10.1038/s41419-020-02800-7)

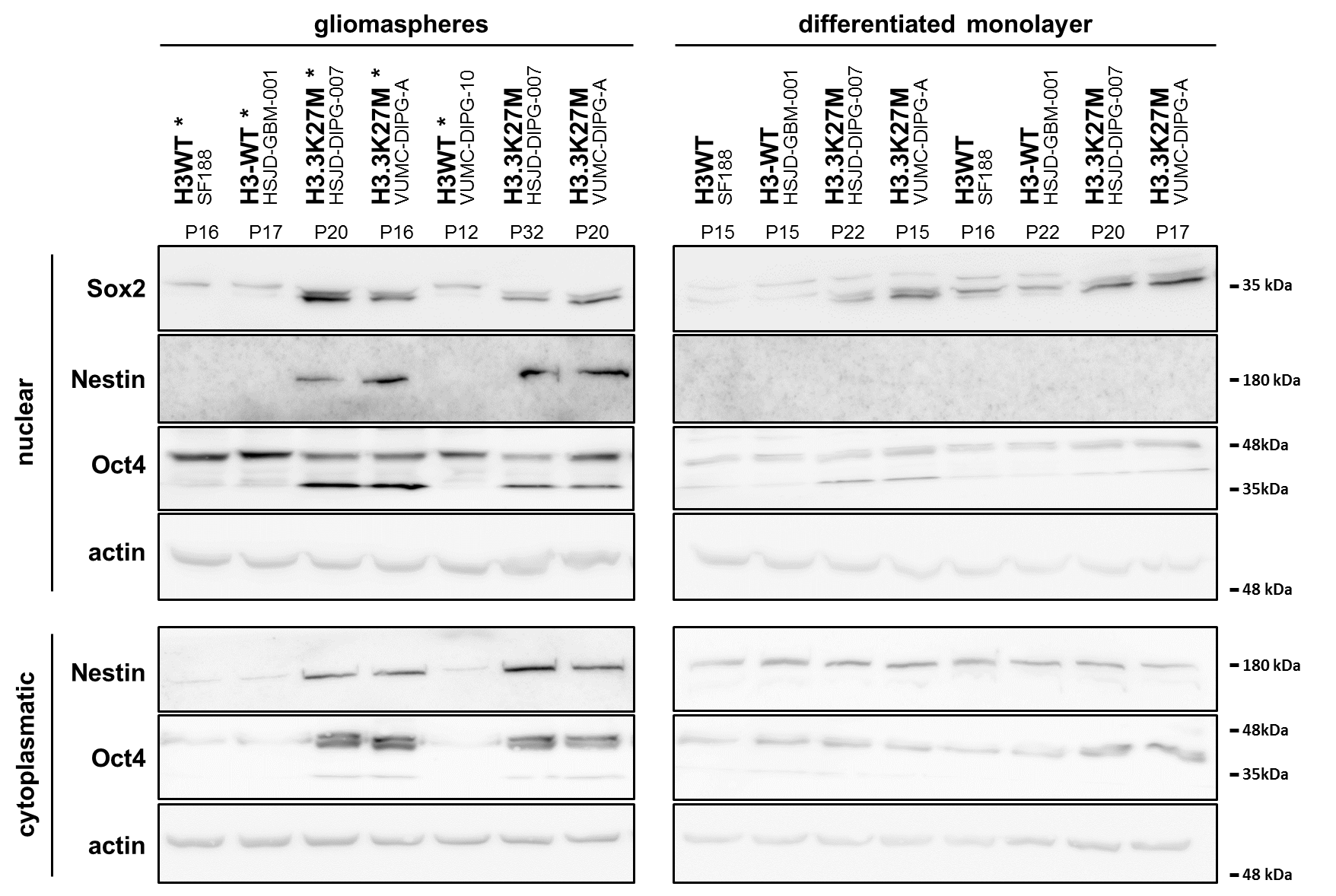

Supplement: Supplementary file 2 — Figure S1 [file 41419_2020_2800_MOESM2_ESM.tif]

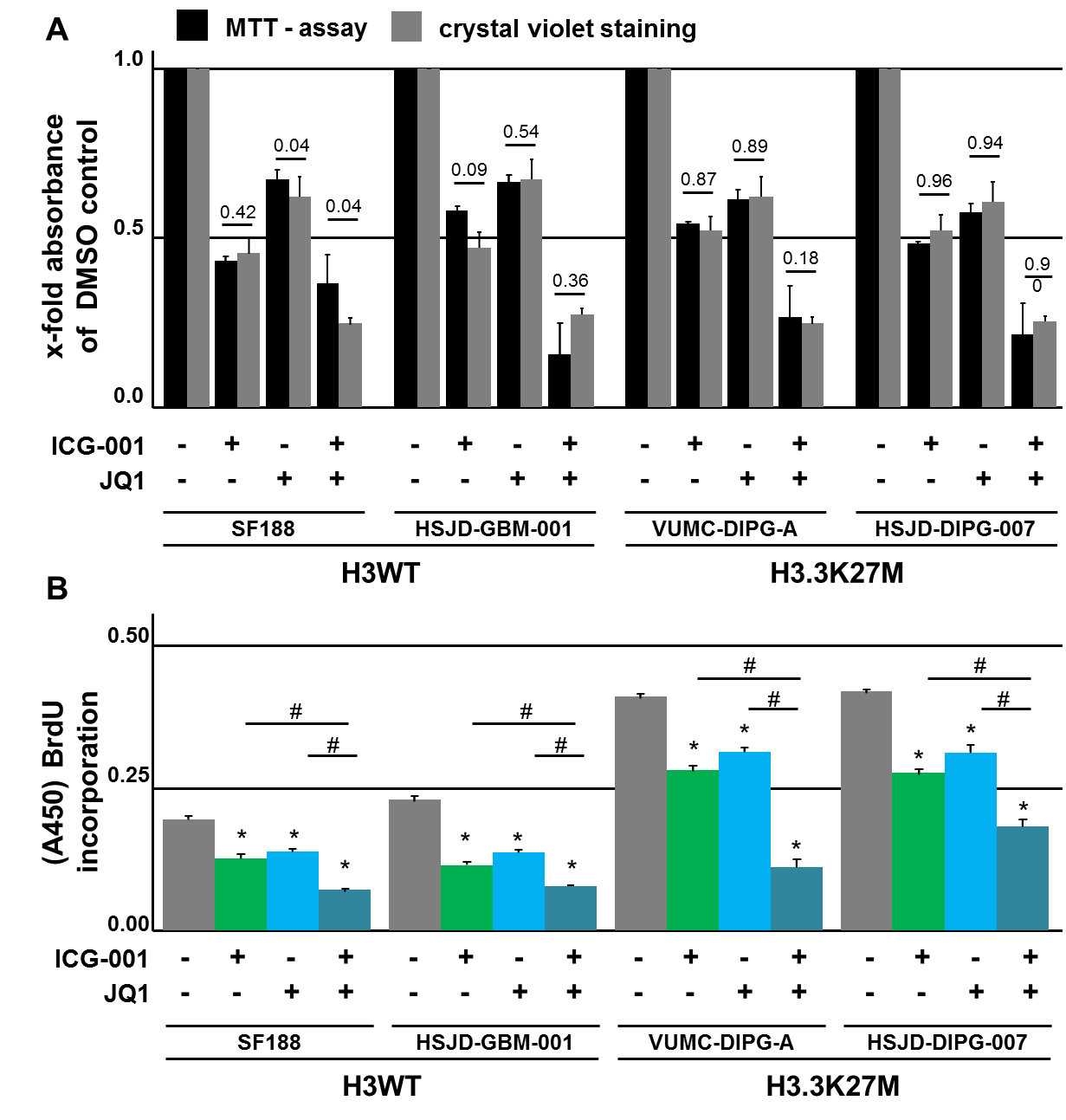

Supplement: Supplementary file 3 — Figure S2 [file 41419_2020_2800_MOESM3_ESM.tif]

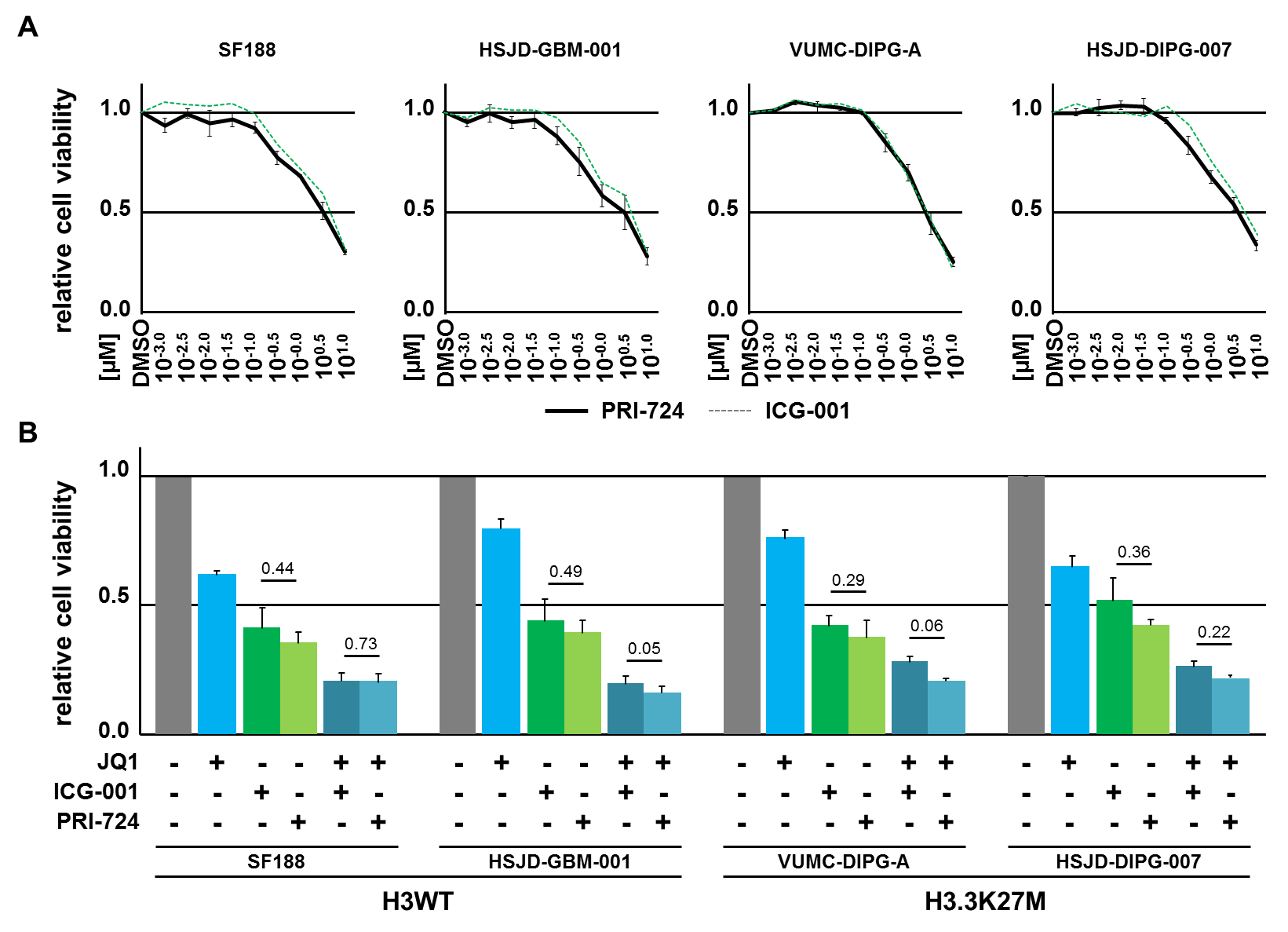

Supplement: Supplementary file 4 — Figure S3 [file 41419_2020_2800_MOESM4_ESM.tif]
