# Supplementary material for: Combined treatment with CBP and BET inhibitors reverses inadvertent activation of detrimental super enhancer programs in DIPG cells
Source: Cell Death Dis. 2020 Aug 21;11(8):673. doi: 10.1038/s41419-020-02800-7 (PMC7442654; doi:10.1038/s41419-020-02800-7)
Supplement: Supplementary file 5 — Supplementary Figures legends [file 41419_2020_2800_MOESM5_ESM.docx]

**Supplemental figure legends**

Supplemental Figure 1│H3K27M-mut-DIPG express higher levels of stemness-associated markers in comparison to H3WT-pedHGG/DIPG cell lines. Expression of the stemness-associated markers Sox2, Nestin and Oct4 in nuclear and cytoplasmatic protein fractions of different cell passages of H3K27M-mut-DIPG and H3WT-pedHGG/DIPG cell lines cultured as gliomaspheres or under differentiation conditions for three days. * Shown in Figure 1, here shown for comparative purpose. ß-actin served as loading control.

Supplemental Figure 2│BET and CBP inhibition inhibit proliferation of H3WT-pedHGG and H3K27M-mut-DIPG cells. (A) MTT-cell viability assay compared to crystal violet staining and (B) BrDU incorporation after 72h incubation of the indicated cell lines with JQ1 and ICG-001. * p < 0.05 with respect to DMSO treated control cells, # p < 0.05 with respect to ICG-001 or JQ1 treated cells, as indicated.

Supplemental Figure 3│Treatment of H3WT-pedHGG and H3K27M-mut-DIPG cells with PRI-724, a derivative of ICG-001, and ICG-001 result in comparable effects. (A) MTT cell viability dilution curves with increasing concentrations of JQ1 and ICG-001, as indicated. (B) MTT cell viability assay after single and combined treatment with ICG-001 or PRI-724 and JQ1 for 72h.
